# Coefficient of Thermal Expansion of AlSi10Mg, 316L Stainless Steel and Ti6Al4V Alloys Made with Laser Powder Bed Fusion

**DOI:** 10.3390/ma18194468

**Published:** 2025-09-25

**Authors:** Selami Emanet, Edem Honu, Kekeli Agbewornu, Evelyn Quansah, Congyuan Zeng, Patrick Mensah

**Affiliations:** 1 Department of Mechanical & Industrial Engineering, Louisiana State University, Baton Rouge, LA 70803, USA; semane1@lsu.edu; 2 Department of Mechanical Engineering, Southern University and A&M College, Baton Rouge, LA 70807, USA; edem.honu@sus.edu (E.H.); kekeli.agbewornu@sus.edu (K.A.); eve100dede@gmail.com (E.Q.); patrick_mensah@subr.edu (P.M.)

**Keywords:** metal additive manufacturing, coefficient of thermal expansion, processing parameters, laser powder bed fusion

## Abstract

This study investigates the coefficient of thermal expansion (CTE) behavior of LPBF-fabricated AlSi10Mg, 316L stainless steel, and Ti-6Al-4V alloys, focusing on the influence of laser power, scanning speed, and annealing. AlSi10Mg exhibits the highest CTE, but its thermal expansion is highly sensitive to porosity and balling defects. 316L stainless steel shows moderate and stable CTE values, with minimal changes after annealing due to its dense microstructure. Ti-6Al-4V has the lowest CTE, with annealing improving expansion. Higher laser power (100 W) improves fusion, reduces porosity, and stabilizes CTE, while lower power (50 W) increases defects, particularly in AlSi10Mg and Ti-6Al-4V. Scanning speed significantly influences porosity, with 0.4 m/s providing an optimal balance between melt efficiency and defect minimization. Annealing enhances CTE uniformity by reducing the lack of fusion defects and refining grain structures, with the greatest improvements seen in low-laser-energy conditions. These findings provide valuable insights for optimizing LPBF parameters to enhance thermal stability and reliability in aerospace, biomedical, and structural applications.

## 1. Introduction

Over the past decade, metal additive manufacturing (AM) has become a prominent topic in the metal fabrication industry. Particularly, laser powder bed fusion (LPBF), a promising metal AM technique, has garnered significant attention as a high-resolution metal fabrication process with ongoing advancements. LPBF is a powder-based AM technique that produces metallic parts layer-by-layer from a 3D computer-aided design (CAD) model. This technique is also referred to as direct metal laser melting (DMLM) or selective laser melting (SLM) in the literature. During a typical LPBF process, parts are produced by selectively melting and consolidating thin layers of powders using a scanning laser beam guided by CAD data [1,2].

Currently, there is a growing interest in the AM industry in utilizing this technology to produce metal parts with high geometrical complexity, ensuring design freedom. In addition to offering design freedom through complex geometrical designs, the LPBF process provides significant advantages over conventional manufacturing methods. These advantages include reduced manufacturing time and the ability to fabricate a large number of parts without the need for supplementary processes, thereby reducing production lead times [3,4,5,6]. Nonetheless, the primary goal among AM experts is to enhance the efficiency of the LPBF process by producing parts with improved properties and fewer defects. Achieving this goal could significantly reduce the reliance on traditional part production methods such as casting and wrought processes. However, a significant challenge limiting the advancement of LPBF in producing high-quality components with minimal defects and enhanced performance lies in the complex optimization of the numerous interdependent processing parameters involved in the fabrication process and the appropriate post processing technique to optimize part property [7,8,9,10]. During the LPBF process, the fabrication of a component is controlled by a combination of specific processing parameters, including laser power, scanning speed, hatch spacing, and layer thickness. These parameters are carefully selected and optimized to influence melt pool dynamics, microstructural evolution, and overall build quality. The interplay of these parameters significantly influences the thermal history and energy distribution during fabrication, often resulting in spatial variability in the mechanical, morphological, and thermophysical characteristics of the final build. These variations are largely attributed to localized heating and the evolution of temperature-dependent material properties throughout the process [11,12]. Consequently, a systematic investigation of mechanical behavior, microstructural morphology, and thermophysical responses associated with specific parameter combinations is essential for optimizing LPBF-produced parts for specific applications.

Particularly, to assess the evolution of material thermophysical properties, quantitative methods typically used include thermal property testing techniques such as thermal diffusivity, thermal conductivity, specific heat capacity, and coefficient of thermal expansion (CTE) measurements. Among these, CTE assessments are particularly valuable as they provide insights into how a material’s dimensions change with temperature variations. These measurements indicate the fractional change in a material’s size per degree of temperature change under constant strain. In essence, they provide a precise measure of the extent to which a component expands or contracts with each degree of temperature change. This is expressed mathematically in Equation (1) as [13]:

(1) $$\alpha =\;\frac{\Delta L}{L_{0}\Delta T}\;$$

where *α* represents the CTE, Δ*L* refers to the change in length, *L*_0_ is the original length and Δ*T* represents the change in temperature. Accurate prediction of material behavior under varying thermal conditions is essential in engineering design and material selection. Such predictions can provide fundamental insights into how materials respond to thermal loading, enabling designers and engineers to account for thermally induced dimensional changes during structural analyses. Materials characterized by a low CTE generally exhibit superior dimensional stability, making them suitable for applications requiring high precision and minimal thermal deformation. Conversely, materials with higher CTE values undergo more pronounced thermal expansion or contraction, which can be advantageous in systems where flexibility and thermal accommodation are required to maintain functional integrity across a wide temperature range.

Several studies have investigated the influence of varying processing parameter combinations on the thermophysical behavior of metallic materials produced via LPBF, with a particular focus on CTE characterization. For instance, Harrison et al. [14] examined the effect of LPBF process parameters on the thermal expansion behavior of 64Fe-36Ni (Invar36), a material known for its exceptionally low CTE. Their study aimed to determine whether the inherent low thermal expansion characteristic of Invar36 could be retained under different LPBF processing conditions. The study revealed that Invar36 components fabricated via LPBF exhibited even lower CTE values than their conventionally manufactured counterparts, suggesting that the metal AM process may further enhance the material’s dimensional stability. Yakout et al. [15] have also evaluated the CTE of Invar36 and 316L stainless steel components manufactured via the LPBF process. They revealed that 316L stainless steel specimens fabricated at an energy density of 104.2 J/mm^3^ exhibited a higher thermal expansion at lower temperatures compared to counterparts produced at energy densities of 41.7 J/mm^3^ and 156.3 J/mm^3^. These findings highlight the sensitivity of thermophysical behavior to specific energy input conditions during fabrication, underscoring the need for precise control of processing parameters to achieve desirable thermal performance in additively manufactured components.

Hanemann et al. [16] also investigated the effect of alloying elements on the CTE properties of AlSi10Mg + Si alloys. Their study examined two different Si-containing samples: one with 25 wt% Si (processed at a laser power of ≥275 W and scan speeds of ≥1500 mm/s) and another with 50 wt% Si (processed at 400 W and scan speeds of ≥1500 mm/s). The results indicated that increasing Si content significantly reduced the overall CTE, with a 43% reduction observed for AlSi10Mg with a 50 wt% Si. For Ti-6Al-4V, Yakout et al. [17] investigated the interplay between thermal expansion behavior and residual stress development in components fabricated via LPBF. They examined how rapid heating and cooling rates intrinsic to the LPBF process influenced residual stress accumulation and thermal expansion characteristics. The results revealed a non-linear trend wherein increasing the laser energy density initially led to a rise in both thermal expansion and residual stress, followed by a decline beyond a critical energy density threshold. This behavior indicates the complex thermomechanical interactions that govern the final properties of LPBF-fabricated Ti-6Al-4V parts and highlights the importance of energy input optimization to balance dimensional stability and residual stress management.

Nonetheless, while individual studies have examined the CTE of specific materials, a significant gap remains in systematically comparing the CTE behavior of commonly used LPBF alloys, such as AlSi10Mg, 316L stainless steel, and Ti-6Al-4V, across both as-fabricated and annealed conditions. Given the importance of thermal expansion in structural integrity and component reliability, a comprehensive understanding of how these materials respond to different thermal conditions is crucial for optimizing their applications in specific engineering environments such as aerospace. Thus, this study aims to address this gap by investigating the CTE variations in these alloys, providing critical insights into their thermal stability and performance. While prior studies have focused on optimizing LPBF processing parameters to enhance mechanical properties, little attention has been given to how these parameters influence thermophysical behavior, particularly CTE. In fact, Akwaboa et al. [18] and Ni et al. [19] have demonstrated the importance of process parameter optimization in minimizing defects and controlling microstructural evolution in these alloys. These studies identified strategies, such as adjusting the energy input to reduce porosity and internal stress, leading to improved mechanical properties. However, their findings primarily focused on strength, ductility, and fatigue resistance, without addressing the thermal expansion behavior. As previously mentioned, because dimensional stability under thermal fluctuations is a key requirement for high-performance applications, understanding the CTE characteristics of these LPBF-fabricated alloys under different processing and heat treatment conditions is worthwhile.

While individual studies have reported CTE values for LPBF AlSi10Mg, 316L, and Ti-6Al-4V, there is no unified dataset comparing these alloys under consistent fabrication and heat treatment conditions, which limits cross-material design for thermal management in AM applications. To bridge this gap, this study will systematically analyze the thermal expansion characteristics of AlSi10Mg, 316L stainless steel, and Ti-6Al-4V in both as-fabricated and annealed conditions. The insights gained will contribute to a more comprehensive understanding of the thermophysical properties of LPBF-produced alloys, enabling the development of improved processing strategies and design considerations for temperature-sensitive applications.

## 2. Materials and Methods

### 2.1. Sample Preparation

**Figure 1:**
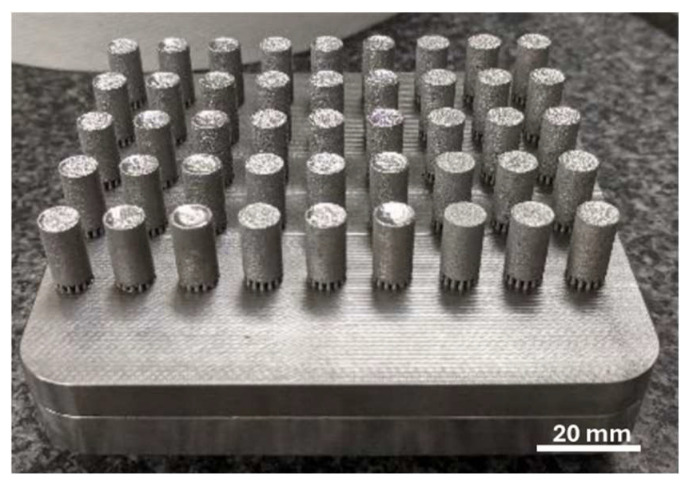
Image showing the shape and size of the as-fabricated 316L stainless steel samples.

This study systematically examined three different alloys: 316L stainless steel, AlSi10Mg, and Ti-6Al-4V. The metal powders used had particle sizes ranging from 25 to 50 μm, which were provided by Concept Laser GmbH (Lichtenfels, Germany), now acquired by GE Additive (West Chester, OH, USA). Laser power and scanning speed, the primary processing parameters considered, were adjusted for each sample. All samples were fabricated using the SLM Concept-Laser Mlab Cusing R LPBF system (Lichtenfels, Germany) located at Louisiana State University (LSU). This system is equipped with a 100 W continuous-wave (CW) fiber laser operating at a wavelength of ~1.07 μm, a scanning speed of up to 7000 mm/s, a layer thickness of 15–50 μm, and a laser spot size of approximately 50 μm. In this study, nine different variations in parameters were examined, using the two primary parameters, namely laser power (50 W, 75 W and 100 W) and laser scanning speed (0.2 m/s, 0.4 m/s and 0.6 m/s), while keeping the rest of the processing parameters constant, specifically hatch space to be 50 μm, and layer thickness to be 25 μm (Table 1). In LPBF, the typical metric used is volumetric energy density (*VED*), usually expressed in J/mm^3^, defined as: $VED=\;\frac{P}{v\cdot h\cdot t}$, where P = laser power (W), v = laser scan speed (mm/s), h = hatch spacing (mm), and t = layer thickness (mm). Based on the processing parameters in this study, a broader range of energy densities, from 66.7 J/mm^3^ to 400 J/mm^3^, was examined (Table 1). According to the literature, the typical laser processing energy density for Ti-6Al-4V, 316L stainless steel, and AlSi10Mg alloy samples ranges from 60 to 120 J/mm^3^ [20,21,22,23,24,25,26]. Therefore, this study’s *VED* covers and goes beyond the optimized laser energy density, given that the goal of this study was to investigate the effect of laser processing parameters on thermophysical properties. To optimize time and cost efficiency, samples of the same material but with varying properties, resulting from different processing parameters, were produced on the same build plate, shown in Figure 1. Each sample was labeled according to its specific processing parameters for easy identification. The sample dimensions were selected based on the standard specifications of the Netzsch Dilatometer for thermal expansion property testing. Specifically, cylindrical samples with a height of 12 mm and a diameter of 6 mm were fabricated for each alloy. To ensure the repeatability and reliability of the test data, three samples were fabricated and tested for each condition.

Annealing heat treatments were performed using a Thomas Scientific FD 1535M furnace (Swedesboro, NJ, USA) for all the fabricated alloys samples, and the heat treatment profiles are listed below in Table 2. The heat treatment profiles were supported by previous studies by other researchers [27,28,29,30,31].

The Netzsch DIL 402C equipment (manufactured by Netzsch Instruments in Selb, Germany), was used to determine the CTE values for each sample. To determine the CTE, the samples were heated in a vacuum-sealed and atmosphere-controlled environment, and the thermal expansion in terms of unit size change in the sample was measured using a highly accurate dilatometer (Netzsch Instruments, Selb, Germany).

### 2.2. Microstructural Analysis

To investigate the evolution of defects, particularly lack of fusion, porosity and balling effects, microstructural and surface morphology analyses were performed on all LPBF-processed and annealed samples using a Phenom ProX scanning electron microscope (SEM) (Phenom-World, Eindhoven, The Netherlands, now part of Thermo Fisher Scientific, Waltham, MA, USA) for surface morphology analysis and an optical microscope (BX60M, Olympus Corporation, Tokyo, Japan) for examining cross-sectional microstructures. For precise microstructural characterization, the samples were etched as follows: the 316 stainless steel samples were etched in a solution of 5 mL HNO_3_ and 15 mL HCl for 20 s; the AlSi10Mg samples were etched using a solution comprising 2.5 vol% HNO_3_, 1 vol% HF, 95 vol% H_2_O, and 1.5 vol% HCl for 20 s; and the Ti-6Al-4V alloy samples were etched in a solution containing 50 mL H_2_O, 25 mL HNO_3_, and 5 mL HF for 20 s. Prior to etching, all sample surfaces were sequentially ground using SiC papers with grit sizes 400, 600, 800, 1000 and 1200 mesh, followed by polishing with diamond suspensions of 6 μm, 3 μm and 1 μm in sequence.

## 3. Results

### 3.1. CTEs for AlSi10Mg Alloy

**Figure 2:**
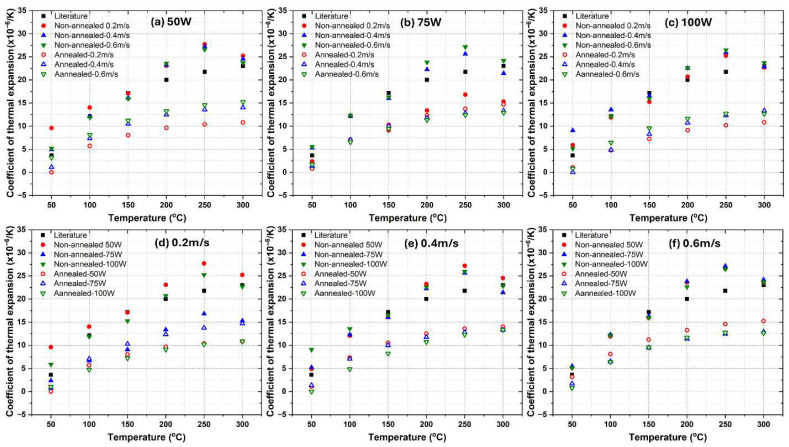
Comparative analysis of the CTEs of as-fabricated and annealed AlSi10Mg alloys under varying processing conditions. (**a**–**c**) illustrate the influence of constant laser power settings (50 W, 75 W and 100 W), while (**d**–**f**) examine the effects of fixed laser scanning speeds (0.2 m/s, 0.4 m/s, and 0.6 m/s).

Figure 2 presents the CTE values for LPBF fabricated AlSi10Mg samples under different processing conditions, comparing both as-fabricated and annealed states. The results are categorized based on variations in laser power (50 W, 75 W and 100 W) and laser scanning speed (0.2 m/s, 0.4 m/s and 0.6 m/s). The findings indicate significant variations in CTE across different temperatures and process conditions. It is worth noting that standard deviations for all the measured data for the three alloys are within ±0.3 × 10^−6^/K, which indicates good measurement repeatability.

The CTE values of LPBF AlSi10Mg alloy were analyzed based on laser power, scan speed, annealing treatment, and comparison with literature data. The first row of subplots (Figure 2a–c) groups the CTE data by laser power (50 W, 75 W, and 100 W), showing that non-annealed samples consistently exhibit higher CTE values than annealed ones across all temperatures. As temperature increases, the CTE values generally rise, but non-annealed samples display more pronounced increases compared to their annealed counterparts. Increasing laser power slightly raises the CTE values in non-annealed samples, particularly at higher temperatures, whereas annealed samples maintain lower and more stable CTE values across different scan speeds and laser powers. Literature values align more closely with non-annealed samples over the testing temperature range [32]. The second row of subplots (Figure 2d–f) organizes the data by scan speed (0.2 m/s, 0.4 m/s and 0.6 m/s) for different laser powers. At lower scan speeds (0.2 m/s), non-annealed samples exhibit higher CTE values with noticeable variations depending on laser power, while annealed samples show a significant reduction in CTE, bringing them apart from literature values. At medium scan speeds (0.4 m/s), non-annealed samples still display relatively high CTE values, but the spread across different laser power levels is smaller than at lower scan speeds, and annealing reduces the CTE, again apart from literature data. At higher scan speeds (0.6 m/s), non-annealed samples continue to exhibit the highest CTE values, which remain close to literature-reported values, whereas annealed samples maintain the lowest and most stable CTE values. In general, annealing consistently reduces CTE values across all laser powers and scan speeds, indicating that it effectively relieves residual stresses and promotes a more thermally stable microstructure. Non-annealed samples produced at higher laser powers (100 W) tend to have slightly lower CTE values than those made at lower powers (50 W), possibly due to differences in microstructure, such as Si phase coarsening or residual stress variations [33]. Increasing the scan speed reduces the CTE of non-annealed samples, likely due to finer microstructural features and higher retained residual stresses, while annealed samples exhibit minimal sensitivity to scan speed, maintaining lower and more stable CTE values. Finally, the CTE increases with temperature for all samples, but the rate of increase is lower in annealed samples, suggesting improved thermal stability. The literature data provides a benchmark, reinforcing the observation that non-annealed samples exhibit thermal expansion behavior comparable to conventionally processed AlSi10Mg alloys, while annealed samples tend to deviate likely due to Si phase coarsening. Notably, when the temperature reached 300 °C, a clear decline in CTE was observed for the non-annealed samples, likely due to dynamic precipitate coarsening and residual-stress relaxation at elevated temperature, as reported in the literature [34,35]. In contrast, the heat-treated samples showed a generally monotonic increase in CTE with increasing temperature.

**Figure 3:**
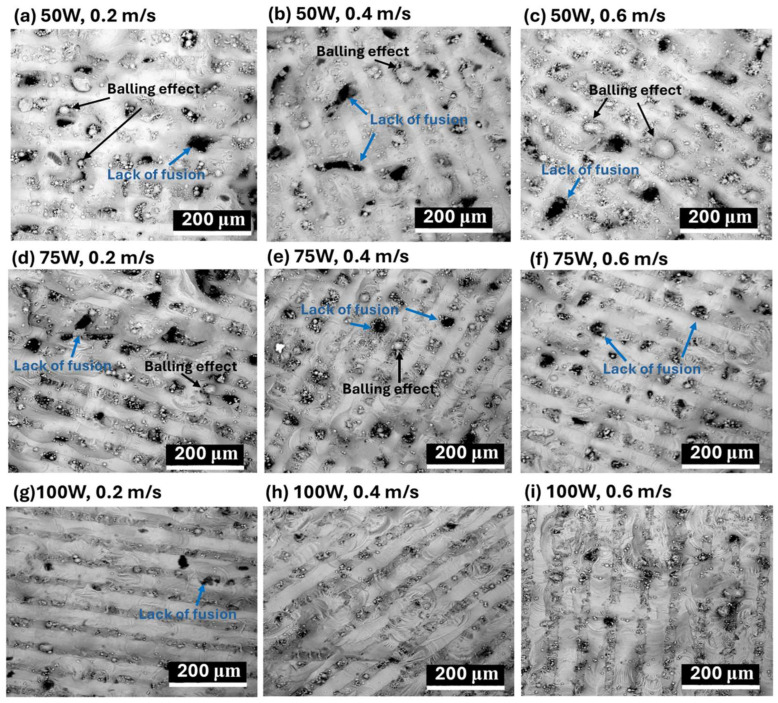
SEM images of the surface morphology of LPBF-processed AlSi10Mg samples under different laser power and scanning speed conditions. The images illustrate the formation of defects such as lack of fusion (blue annotations) and the balling effect (black annotations), which are influenced by process parameters. The conditions are as follows: (**a**–**c**) 50 W laser power with scanning speeds of 0.2 m/s, 0.4 m/s, and 0.6 m/s, respectively; (**d**–**f**) 75 W laser power with scanning speeds of 0.2 m/s, 0.4 m/s, and 0.6 m/s, respectively; and (**g**–**i**) 100 W laser power with scanning speeds of 0.2 m/s, 0.4 m/s, and 0.6 m/s, respectively.

**Figure 4:**
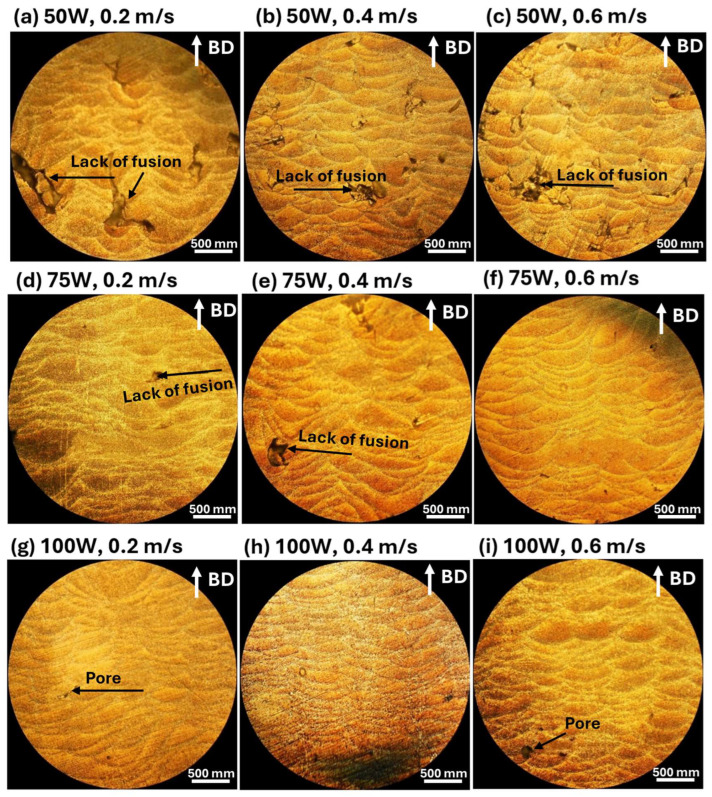
These optical microscope images illustrate the microstructural characteristics of LPBF-fabricated AlSi10Mg samples (non-annealed) produced under different laser power and scanning speed conditions. The images in the first row (**a**–**c**) correspond to samples fabricated with 50 W laser power at scanning speeds of 0.2 m/s (**a**), 0.4 m/s (**b**), and 0.6 m/s (**c**). The second row (**d**–**f**) represents samples processed at 75 W, with scanning speeds of 0.2 m/s (**d**), 0.4 m/s (**e**), and 0.6 m/s (**f**). The third row (**g**–**i**) shows samples manufactured at 100 W, with respective scanning speeds of 0.2 m/s (**g**), 0.4 m/s (**h**), and 0.6 m/s (**i**). The build direction (BD) is indicated by the white arrows in each image.

The impact of laser power and scanning speed on the surface morphology and microstructures of LPBF-fabricated AlSi10Mg samples is illustrated in Figure 3 (SEM images) and Figure 4 (optical microscope images).

The influence of laser processing parameters on surface morphology is evident in Figure 3 (SEM images), where variations in laser power (50 W, 75 W, 100 W) and scanning speed (0.2 m/s, 0.4 m/s, 0.6 m/s) significantly affect defect formation. At 50 W, severe porosity and balling effects are observed, particularly at lower scanning speeds (Figure 3a–c). Increasing the laser power to 75 W (Figure 3d–f) reduces these defects slightly, but pores remain visible, especially at moderate scanning speeds. At 100 W (Figure 3g–i), porosity and balling effects are minimized, leading to a smoother and more continuous surface morphology, indicating improved fusion between layers. Scanning speed also plays a crucial role, with lower speeds (0.2 m/s, Figure 3a,d,g)) causing excessive energy input, leading to large molten pools and increased porosity due to keyhole formation. At moderate scanning speeds (0.4 m/s, Figure 3b,e,h), a better balance between energy input and material consolidation is achieved, reducing both balling effects and porosity. However, at higher scanning speeds (0.6 m/s, Figure 3c,f,i), insufficient fusion occurs, increasing balling effects, especially at lower laser powers, leading to an overall rougher and more defective surface morphology.

The influence of laser processing parameters on microstructures is evident in Figure 4 (Optical Microscope Images), which highlights melt pool morphology and defect distribution under different conditions. At 50 W (Figure 4a–c), melt pools with significant porosity and cracks are observed, particularly at higher scanning speeds (Figure 4c). Increasing laser power to 75 W (Figure 4d–f improves fusion, forming more uniform melt pool structures, although some defects remain, especially at lower scanning speeds. At 100 W (Figure 4g–i), melt pool structures become well-defined and continuous, minimizing defects and resulting in more homogeneous microstructures. Scanning speed also affects melt pool formation, with lower speeds (0.2 m/s, Figure 4a,d,g) generating larger melt pools due to prolonged laser-material interaction, leading to higher thermal input and increased risk of cracks and porosity. At moderate scanning speeds (0.4 m/s, Figure 4b,e,h), melt pools become more uniform, achieving a balance between energy input and material solidification. At higher scanning speeds (0.6 m/s, Figure 4c,f,i), melt pools appear smaller and more refined, but insufficient fusion can result in the presence of pores and incomplete bonding, highlighting the importance of optimizing processing parameters for defect minimization.

A lower laser power (50 W) results in increased defects such as porosity and balling effects, especially at higher scanning speeds, while increasing laser power to 75 W or 100 W improves melt pool uniformity and reduces surface defects, particularly at moderate scanning speeds (0.4 m/s). Slow scanning speeds (0.2 m/s) cause excessive heat input, leading to larger melt pools, increased porosity, and cracks, whereas high scanning speeds (0.6 m/s) create smaller melt pools with insufficient fusion, increasing balling effects. Therefore, optimizing laser power and scanning speed is essential for controlling surface morphology and microstructural integrity in LPBF-fabricated AlSi10Mg samples. The best balance is achieved at a moderate laser power (75–100 W) and intermediate scanning speeds (0.4 m/s), where melt pools are well-formed, and defects such as porosity and balling effects are minimized.

Clearly, the CTEs of LPBF-fabricated AlSi10Mg samples are strongly influenced by the microstructural features observed in Figure 3 and Figure 4. Variations in laser power and scanning speed directly affect the porosity, melt pool characteristics, and microstructural defects, which in turn impact their thermal expansion behavior. The discussion below explores how these microstructural features correlate with the CTE trends observed in Figure 2.

Porosity, particularly prevalent at low laser power (50 W) and high scanning speeds (0.6 m/s) (Figure 3a–c and Figure 4a–c), reduces material density and disrupts metallic continuity. While porosity typically reduces mechanical properties, it does not directly lead to lower CTE values. Instead, as-fabricated samples with the presence of residual stresses and microstructural inhomogeneity, yield higher CTE values. Figure 2 confirms this trend, where as-fabricated samples with high porosity and defects generally exhibit higher thermal expansion. However, at higher laser power (100 W) and moderate scanning speeds (0.4 m/s) (Figure 3h and Figure 4h), the microstructure is more continuous with fewer defects, leading to a more stable CTE. After annealing, porosity remains largely unchanged, but residual stresses are relieved, grain coarsening occurs [36], and microstructure homogenization yields lower CTE values in all conditions. Balling effects, observed at low laser power (50 W) and high scanning speeds (Figure 3a–c), disrupt material continuity, contributing to residual stress accumulation and microstructural inhomogeneity, which, however, leads to increased CTE values. Similarly, insufficient fusion at very high scanning speeds (0.6 m/s, Figure 3c,f,i) results in poor interlayer bonding and internal stress buildup, further raising the CTE of as-fabricated samples. The CTE results in Figure 2f confirm this, where non-annealed samples at 0.6 m/s exhibit higher CTE values due to retained stress. After annealing, stress relief and microstructural homogenization lead to lower CTE values. Melt pool characteristics strongly influence CTE behavior. Larger, well-defined melt pools at higher laser power (75 W and 100 W, Figure 4d–i) improve interlayer bonding and microstructural continuity, resulting in more uniform thermal expansion. In as-fabricated samples, these characteristics contribute to higher CTE values due to retained stress. Conversely, at lower laser power (50 W, Figure 4a–c), irregular melt pools create weak interlayer bonding, leading to even higher CTE values due to localized stress variations. After annealing, the microstructure undergoes grain coarsening and stress relaxation, significantly reducing CTE values closer to literature data.

A strong correlation exists between microstructure and CTE trends across Figure 2, Figure 3 and Figure 4. Higher porosity and balling effects, which are prevalent at low laser power and high scanning speeds, introduce microstructural defects and stress buildup, resulting in higher CTE values in as-fabricated samples. In contrast, well-formed melt pools at higher laser power and moderate scanning speeds enhance interlayer fusion, leading to relatively lower as-fabricated CTE values. Annealing consistently reduces CTE values by relieving stress, promoting grain growth, and refining microstructural homogeneity. Cracks and insufficient fusion further contribute to higher CTE values in non-annealed samples, but these effects are mitigated post-annealing. This underscores the importance of post-processing in stabilizing the thermal expansion behavior of LPBF-fabricated AlSi10Mg. The optimal CTE performance is achieved at 100 W laser power and 0.4 m/s scanning speed, where defects are minimized, and melt pools are well-formed. Annealing further refines the microstructure, reducing residual stress and lowering CTE values, bringing them somehow apart from literature benchmarks. Further studies are needed to explain this.

### 3.2. CTEs for 316L Stainless Steel

**Figure 5:**
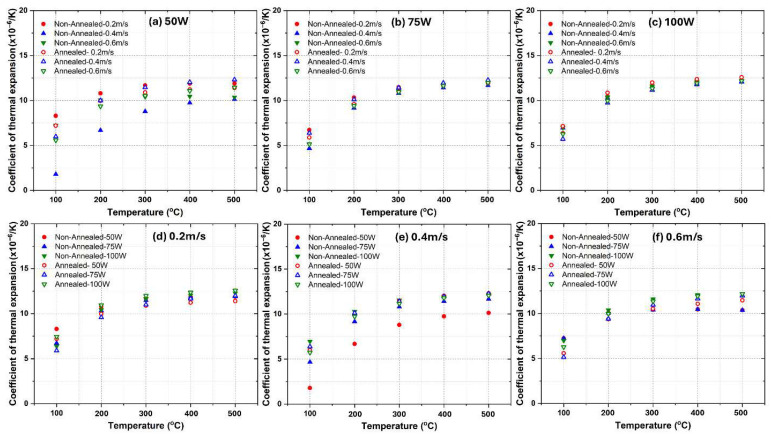
Comparative analysis of the CTEs of as-fabricated and annealed 316L stainless steel under varying processing conditions. (**a**–**c**) illustrate the influence of constant laser power settings (50 W, 75 W, and 100 W), while (**d**–**f**) examine the effects of fixed laser scanning speeds (0.2 m/s, 0.4 m/s, and 0.6 m/s).

Figure 5 demonstrates the CTEs of 316L stainless steel fabricated via LPBF process under varying laser power (50 W, 75 W, and 100 W) and scanning speeds (0.2 m/s, 0.4 m/s, and 0.6 m/s) in both as-fabricated and annealed states. The results provide key insights into the influence of processing parameters and post-annealing treatments on the thermal expansion behavior of 316L stainless steel.

The effect of laser power on the CTEs is displayed across different power levels (Figure 5a–c). At 50 W (Figure 5a), as-fabricated samples exhibit lower CTE values across all temperatures. After annealing, CTE values increase and stabilize, while also reducing variability among different scanning speeds. A similar trend is observed at 75 W (Figure 5b), where the as-fabricated samples have lower CTE values, and annealed samples show higher and more uniform CTE. The difference between scanning speeds diminishes after annealing, suggesting microstructural refinement. At 100 W (Figure 5c), both as-fabricated and annealed samples exhibit relatively stable CTE values. However, the annealing effect is less pronounced, likely due to an improved initial microstructure resulting from the higher energy input.

The effect of scanning speed on the CTEs is demonstrated across different conditions (Figure 5d–f). At 0.2 m/s (Figure 5d), as-fabricated samples exhibit lower CTE values, likely due to high residual stress from excessive heat input. After annealing, CTE increases, confirming the role of heat treatment in stress relaxation. At 0.4 m/s (Figure 5e), moderate scanning speeds yield a more balanced CTE response except the sample made using 50 W, with as-fabricated samples showing less variation and annealed samples stabilizing CTE across temperatures. The difference between laser power settings is minimal after annealing, highlighting improved thermal expansion consistency. At 0.6 m/s (Figure 5f), higher scanning speeds result in lower as-fabricated CTE values, likely due to reduced heat accumulation leading to finer microstructures with more residual stress. Annealed samples show an increase in CTE, though the improvement is slightly less pronounced compared to lower scanning speeds.

The general trends in the CTE behavior reveal that as-fabricated samples consistently exhibit lower CTE values across all conditions. Annealing consistently increases CTE. Higher laser power (i.e., 100 W) results in more stable CTE behavior. Moderate scanning speeds (0.4 m/s) (except the case of 50 W) provide the most consistent thermal expansion performance across different laser powers, indicating an optimal energy input balance. In contrast, higher scanning speeds (0.6 m/s) result in slightly lower as-fabricated CTE values, likely due to finer microstructures and unrelieved residual stress.

**Figure 6:**
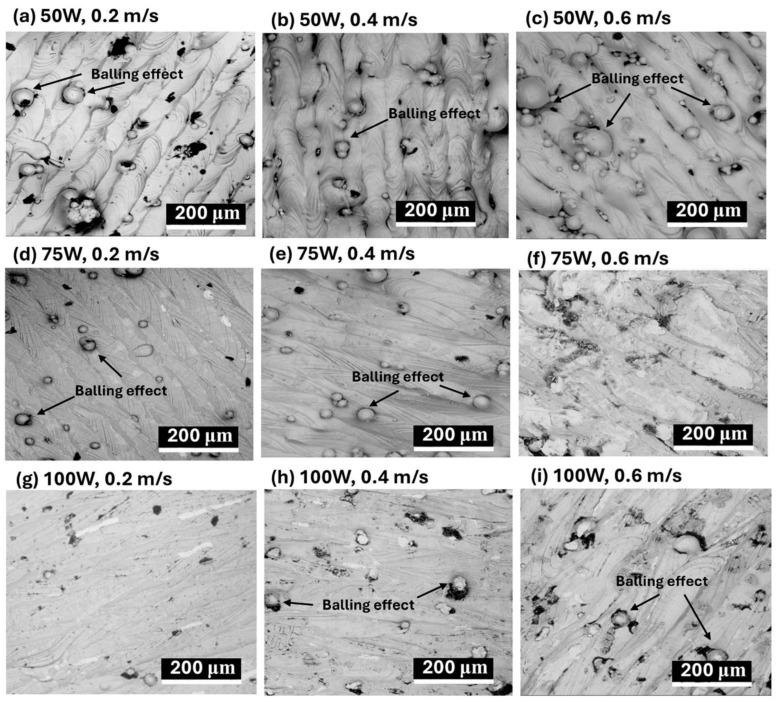
SEM images showing the surface morphology of 316L stainless steel samples fabricated using the laser powder bed fusion (LPBF) process under varying laser power (50 W, 75 W, and 100 W) and scanning speeds (0.2 m/s, 0.4 m/s, and 0.6 m/s). The top row (**a**–**c**) corresponds to 50 W, the middle row (**d**–**f**) to 75 W, and the bottom row (**g**–**i**) to 100 W, with scanning speeds increasing from left to right (0.2 m/s, 0.4 m/s, and 0.6 m/s). Notable surface defects such as pores, balling effects, cracks, and spattering particles are observed, with their severity influenced by processing conditions.

**Figure 7:**
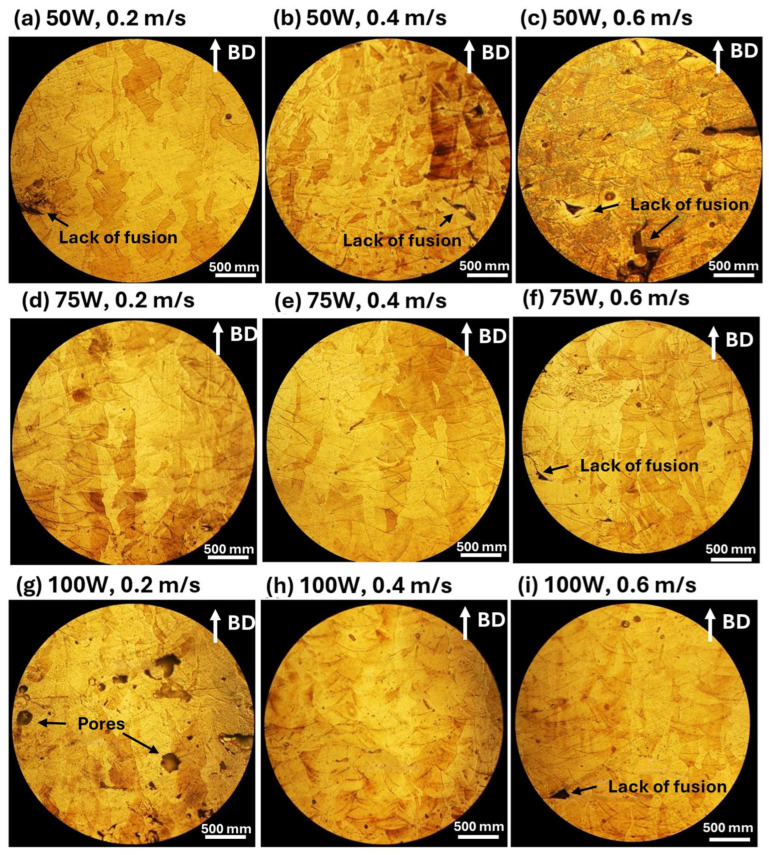
Optical microscopy images of the cross-sectional surface morphology of as-fabricated LPBF-processed 316L stainless steel samples under different laser power and scanning speed conditions. The images reveal defect formations such as lack of fusion and porosity, which are influenced by process parameters. The process conditions are as follows: (**a**–**c**) 50 W laser power with scanning speeds of 0.2 m/s, 0.4 m/s, and 0.6 m/s, respectively; (**d**–**f**) 75 W laser power with scanning speeds of 0.2 m/s, 0.4 m/s, and 0.6 m/s, respectively; and (**g**–**i**) 100 W laser power with scanning speeds of 0.2 m/s, 0.4 m/s, and 0.6 m/s, respectively. The build direction (BD) is indicated by the white arrows in each image.

**Figure 8:**
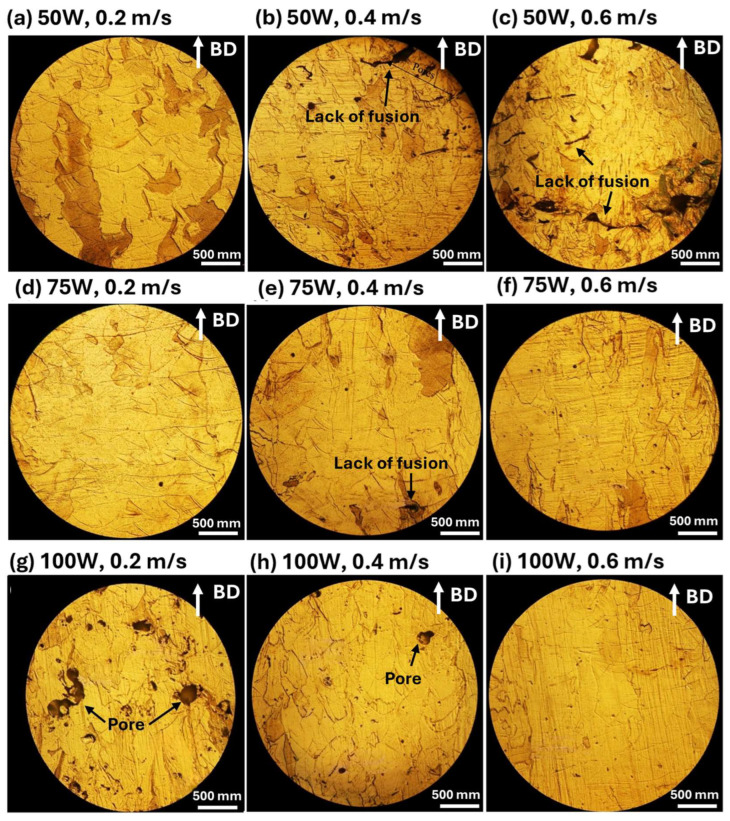
Optical microscopy images of the cross-sectional surface morphology of annealed LPBF-processed 316L stainless steel samples under different laser power and scanning speed conditions. The images reveal defect formations such as lack of fusion and porosity, as well as microstructural changes after annealing. The process conditions are as follows: (**a**–**c**) 50 W laser power with scanning speeds of 0.2 m/s, 0.4 m/s, and 0.6 m/s, respectively; (**d**–**f**) 75 W laser power with scanning speeds of 0.2 m/s, 0.4 m/s, and 0.6 m/s, respectively; and (**g**–**i**) 100 W laser power with scanning speeds of 0.2 m/s, 0.4 m/s, and 0.6 m/s, respectively. The build direction (BD) is indicated by the white arrows in each image.

To have a better understanding of the variation behaviors of as-fabricated and annealed LPBF 316L stainless steel, surface morphology and microstructures of the alloy samples were examined. And the results were shown in Figure 6, Figure 7 and Figure 8.

Figure 6 illustrates the surface morphology of LPBF-processed as-fabricated 316L stainless steel samples. These images provide insight into the melt pool formation, defect distribution, and surface quality under different laser power (50 W, 75 W, 100 W) and scanning speed (0.2 m/s, 0.4 m/s, 0.6 m/s) conditions. Lack of fusion defects are predominantly observed in lower laser power conditions (50 W) (Figure 6a–c), arising due to insufficient melting and weak inter-layer bonding, which leads to voids within the structure. These defects become more pronounced at higher scanning speeds (0.6 m/s) due to reduced energy input per unit area. Porosity is more evident at low energy input (50 W, 0.6 m/s), where lack of fusion pores form, while higher energy density (100 W, lower speeds) results in gas-induced pores, as seen in Figure 6g,h (100 W, 0.2 m/s, 0.4 m/s). The fusion between adjacent layers appears more uniform under optimal conditions (75 W, moderate speeds like 0.4 m/s), with Figure 6d,e (75 W, 0.2–0.4 m/s) showing improved bonding and fewer visible defects. However, excessive energy input (100 W, 0.2 m/s) leads to pore formation due to excessive vaporization and keyhole instability. The build direction (BD), indicated by arrows, influences defect distribution, suggesting that some areas experience directional solidification effects, which impact microstructural uniformity.

Figure 7 represents the cross-sectional microstructures of the as-fabricated 316L stainless steel samples, highlighting defect formation, porosity, and melt pool characteristics. Similar findings are observed in Figure 6. Lack of fusion defects are predominantly observed at lower laser power (50 W) (Figure 7a–c) due to insufficient melting and weak inter-layer bonding, resulting in voids. These defects worsen at higher scanning speeds (0.6 m/s) due to reduced energy input per unit area. Porosity is more pronounced in low-energy conditions (50 W, 0.6 m/s), while higher energy density (100 W, lower speeds) leads to gas-induced pores, as seen in Figure 7g,h (100 W, 0.2–0.4 m/s). Fusion between adjacent layers improves under optimal conditions (75 W, moderate speeds like 0.4 m/s), with Figure 7d,e (75 W, 0.2–0.4 m/s) showing better bonding and fewer defects. However, excessive energy input (100 W, 0.2 m/s) causes pore formation due to vaporization and keyhole instability.

Figure 8 displays the cross-sectional microstructure of annealed 316L stainless steel samples, showing the impact of post-processing heat treatment on porosity, defect reduction, and microstructural evolution. The annealing process promotes diffusion, leading to the partial healing of lack of fusion defects. Despite annealing, pores remain, particularly in samples processed at 100 W, 0.2 m/s (Figure 8g), where some coalesce or expand due to diffusion-driven growth. Annealing also induces grain growth and enhances microstructural homogeneity, transforming the columnar grain structure of the as-fabricated state into a more equiaxed grain structure. The BD, indicated in the images, continues to influence grain growth after heat treatment, though the layered structure characteristic of LPBF remains partially visible.

The microstructural characteristics of LPBF-processed 316L stainless steel, including lack of fusion defects, porosity, and grain morphology, play a crucial role in determining its CTE behavior. The attached CTE results, plotted against temperature for different laser powers (50 W, 75 W, and 100 W) and scanning speeds (0.2 m/s, 0.4 m/s, and 0.6 m/s), show a clear correlation with the microstructures observed in the SEM and OM images.

In the as-fabricated condition, samples processed at lower laser power (50 W) and higher scanning speed (0.6 m/s) exhibit significantly lower CTE values at lower temperatures due to the presence of lack of fusion defects, which act as voids, reducing the effective material density and limiting thermal expansion. As temperature increases, CTE values rise, likely due to the closure of micro voids and defect relaxation during expansion. In contrast, samples processed at higher laser power (100 W) and lower scanning speeds (0.2–0.4 m/s) show more stable and higher CTE values, as improved melting enhances layer bonding and minimizes void-induced thermal resistance. Porosity remains a major factor affecting the thermal expansion response, as observed in the microstructures. Samples with higher porosity, such as those processed at 100 W and 0.2 m/s, exhibit slightly lower CTE values because gas-induced pores act as thermal expansion inhibitors, disrupting the uniform expansion of the matrix. However, annealed samples show higher and more consistent CTE values, indicating that heat treatment reduces the effects of porosity and enhances material homogeneity. This effect is particularly noticeable in 100 W samples, where annealing helps stabilize the thermal expansion behavior. In the as-fabricated state, the fine columnar grain structures in LPBF-processed 316L stainless steel result in a relatively anisotropic thermal expansion behavior, with variations in CTE observed across different temperature ranges. After annealing, grain growth and recrystallization lead to the formation of a more equiaxed grain structure, enhancing isotropic thermal expansion and resulting in a more uniform CTE response. This effect is particularly evident in annealed samples processed at 75 W and 100 W, where grain refinement and homogenization contribute to a more stable expansion behavior over a wide temperature range.

Annealed samples consistently exhibit higher and more uniform CTE values compared to as-fabricated samples, highlighting the role of defect reduction and microstructural homogenization in improving thermal expansion behavior. The most pronounced improvements are observed in 50 W and 75 W samples, where annealing effectively reduces the influence of lack of fusion defects. At higher laser power (100 W), the difference between as-fabricated and annealed samples is smaller, suggesting that the higher energy input during fabrication already promotes a more stable microstructure with fewer defects affecting CTE.

### 3.3. CTEs for Ti-6Al-4V Alloy

**Figure 9:**
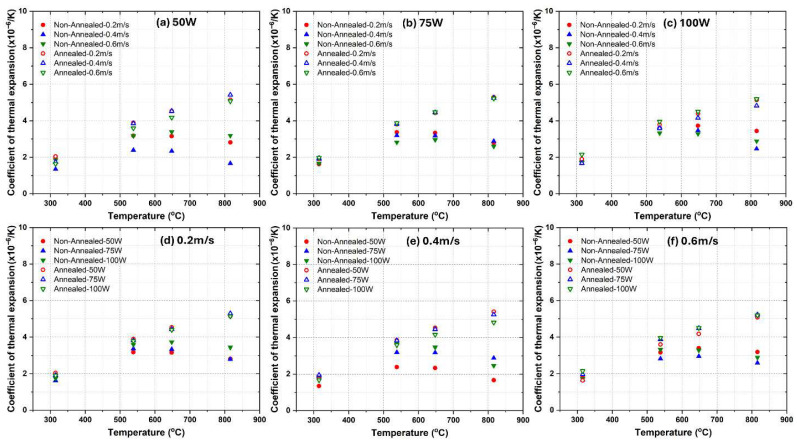
CTEs of LPBF-processed Ti-6Al-4V alloys in both as-fabricated and annealed states, plotted as a function of temperature. The data is categorized based on laser power (50 W, 75 W, and 100 W) and scanning speed (0.2 m/s, 0.4 m/s, and 0.6 m/s). Panels (**a**–**c**) display the CTE trends for different laser powers, while (**d**–**f**) categorize the results by scanning speed.

The CTE results presented in Figure 9 illustrate the thermal expansion behavior of Ti-6Al-4V alloys processed by LPBF in both as-fabricated and annealed conditions. The data is categorized based on laser power (50 W, 75 W, 100 W) and scanning speed (0.2 m/s, 0.4 m/s, and 0.6 m/s), showing variations in CTE as a function of temperature (300–900 °C).

CTE varies with both laser power and scan speed (Figure 9a–f). Laser power (Figure 9a–c): At 50 W, as-fabricated samples show lower, more scattered CTE, especially at high temperatures, while annealed samples are higher and more stable across the range. At 75 W, as-fabricated CTE becomes more uniform, while annealed remains consistently higher. At 100 W, as-fabricated CTE is highest and most stable, while annealing yields only a slight further increase. Overall, higher power yields higher and more uniform CTE, with annealing improving stability most at lower power. Scan speed (Figure 9d–f): At 0.2 m/s, as-fabricated 50 W shows the lowest CTE, whereas 100 W is more stable and higher. Annealing increases CTE, especially at 50/75 W. At 0.4 m/s, CTE is more scattered across powers, while annealed samples are slightly higher and more stable, with larger as-fabricated/annealed gaps than at 0.2 m/s. At 0.6 m/s, as-fabricated CTE becomes consistent once again, while annealing raises and homogenizes values.

**Figure 10:**
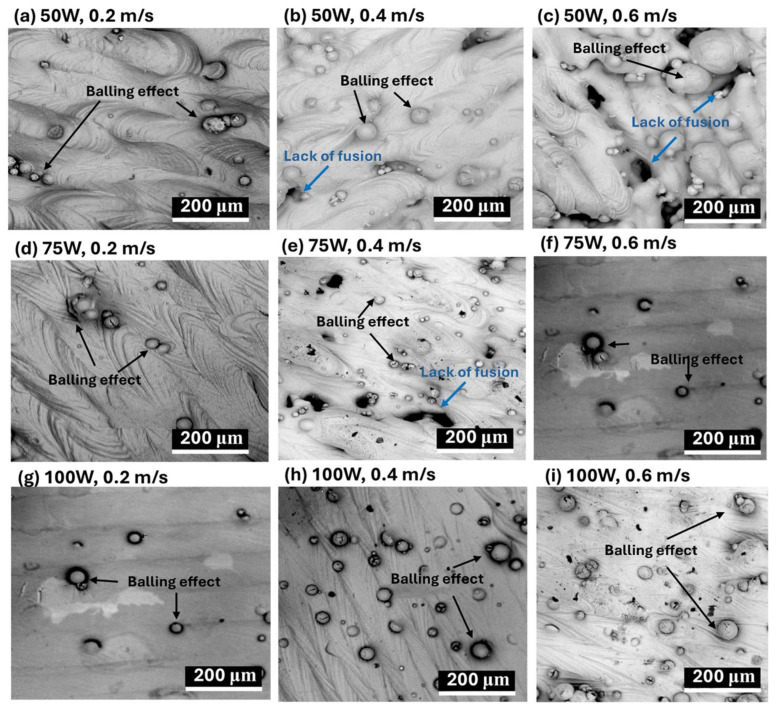
SEM images showing the surface morphology of Ti-6Al-4V alloy samples fabricated using the LPBF method under varying laser power and scanning speed conditions. The images reveal the presence of balling effects and lack of fusion defects, which are influenced by processing parameters. The conditions are as follows: (**a**–**c**) 50 W laser power at scanning speeds of 0.2 m/s, 0.4 m/s, and 0.6 m/s, respectively; (**d**–**f**) 75 W laser power at scanning speeds of 0.2 m/s, 0.4 m/s, and 0.6 m/s, respectively; (**g**–**i**) 100 W laser power at scanning speeds of 0.2 m/s, 0.4 m/s, and 0.6 m/s, respectively.

**Figure 11:**
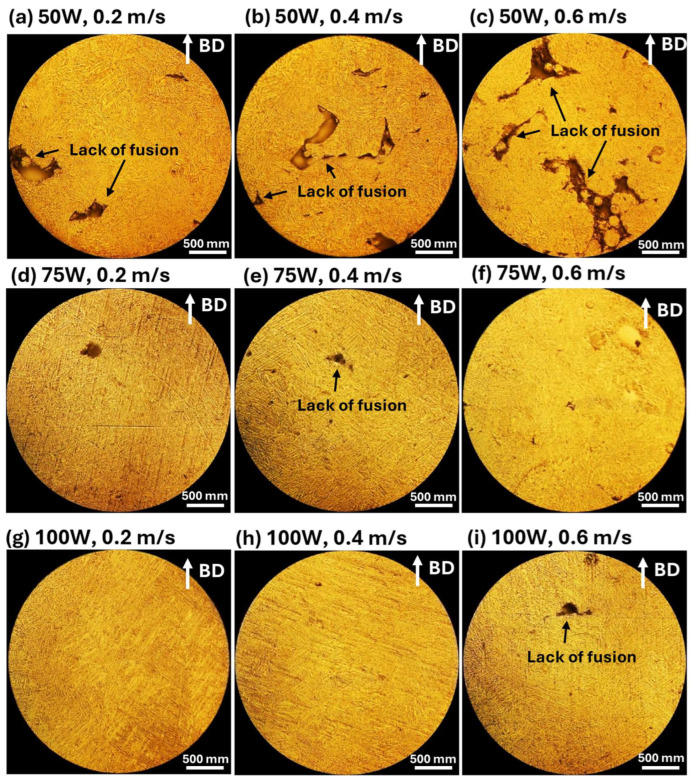
Optical microscopy images of the cross-sections of as-fabricated LPBF Ti-6Al-4V alloy under varying laser power (50 W, 75 W, and 100 W) and scanning speed (0.2 m/s, 0.4 m/s, and 0.6 m/s). The build direction (BD) is indicated by white arrows in each image. Lack of fusion defects, caused by insufficient melting and poor inter-layer bonding, are more prevalent at low laser power (50 W) and high scanning speeds (0.6 m/s) (**a**–**c**). As laser power increases to 75 W (**d**–**f**) and 100 W (**g**–**i**), lack of fusion defects are reduced, with 100 W samples exhibiting the most uniform microstructure and minimal defects.

**Figure 12:**
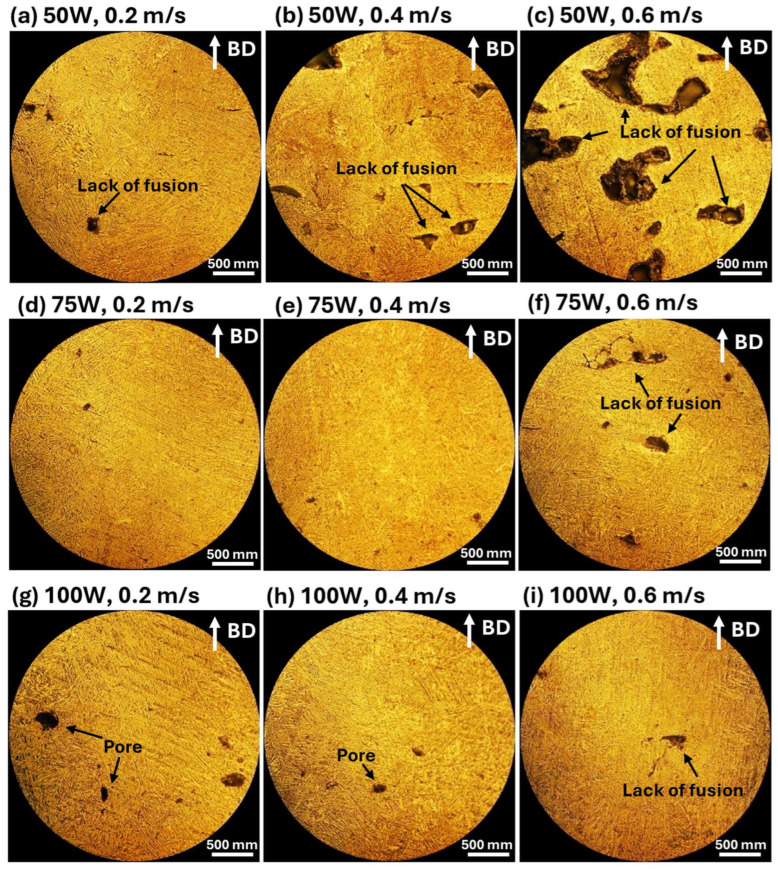
Optical microscopy images of the cross-sections of annealed LPBF Ti-6Al-4V alloy under varying laser power (50 W, 75 W, 100 W) and scanning speed (0.2–0.6 m/s). The build direction (BD) is indicated by white arrows. Lack of fusion defects, prominent in 50 W and high-speed samples (**a**–**c**), are reduced compared to the as-fabricated state due to annealing. Pores, mainly observed in 100 W, low-speed samples (**g**,**h**), suggest gas entrapment from keyhole effects.

Similar to LPBF AlSi10Mg and 316L stainless steel, microstructural tests were conducted to better understand the CTE variation behavior of LPBF Ti-6Al-4V alloy. The surface morphology of the as-fabricated Ti-6Al-4V alloy and the cross-sectional views of the as-fabricated and annealed Ti-6Al-4V alloys are shown in Figure 10, Figure 11 and Figure 12, respectively.

Figure 10 provides a detailed visualization of the surface morphology of Ti-6Al-4V samples fabricated using the LPBF process, highlighting the effects of laser power (50 W, 75 W, and 100 W) and scanning speed (0.2 m/s, 0.4 m/s, and 0.6 m/s) on defect formation. The observed surface features primarily include balling effects and lack of fusion defects, both of which are highly dependent on the energy density supplied during the LPBF process. The surface morphology of LPBF-processed Ti-6Al-4V alloy is significantly influenced by laser power and scanning speed. At low laser power (50 W), a pronounced balling effect, characterized by spherical formations due to insufficient energy input and poor melt pool wetting, is evident (Figure 10a–c). This effect intensifies at higher scanning speeds, accompanied by severe lack of fusion defects resulting from incomplete melting and poor interlayer bonding, negatively impacting mechanical properties. Medium laser power (75 W) conditions reduce balling and minimize lack of fusion defects at moderate scanning speeds (0.4 m/s), indicating improved melt stability. However, at higher scanning speeds (0.6 m/s), instability and surface roughness increase (Figure 10d–f). High laser power (100 W) leads to excessive vaporization, causing gas-induced pores and keyhole defects, particularly at lower scanning speeds (0.2–0.4 m/s, Figure 10g,h). At high scanning speed (0.6 m/s), melt pool breakup and severe balling further degrade surface quality, increasing irregularities and reducing material integrity (Figure 10i).

Figure 11 demonstrates the optical microscope for the cross-sections of as-fabricated Ti-6Al-4V alloy. The as-fabricated Ti-6Al-4V samples exhibit varying microstructural defects depending on laser power and scanning speed. Lack of fusion defects are most prominent in low-power (50 W) and high-speed (0.6 m/s) samples (Figure 11a–c) due to insufficient energy input, resulting in incomplete melting and weak interlayer bonding. Some lack of fusion defects remain visible in 75 W (Figure 11d) and 100 W (Figure 11i) samples, though less severe than in 50 W conditions. Microstructural homogeneity improves with higher laser power (100 W) and low/moderate scanning speeds (0.2 m/s, 0.4 m/s, Figure 11g,h), where defects are significantly reduced, suggesting better melt pool stability and enhanced densification. Porosity is minimal in the 50 W and 75 W samples, as these parameters primarily result in lack of fusion rather than gas-induced voids. However, 100 W samples show more pronounced porosity (Figure 11g–i), especially at low speeds (0.2 m/s, Figure 11g), due to increased keyhole effects and vaporization-induced voids. Overall, 50 W, 0.6 m/s exhibits the most severe lack of fusion defects, 75 W, 0.4 m/s provides an optimal balance with minimal defects, and 100 W, 0.2 m/s results in gas-induced porosity from excessive energy input.

Figure 12 demonstrates the optical microscope for the cross-sections of annealed Ti-6Al-4V alloy. Annealing significantly improves the microstructure of LPBF Ti-6Al-4V alloy, refining grain structures, and modifying porosity distribution. Microstructural diffusion during annealing reduces the visibility of lack of fusion defects, with previously severely affected samples (50 W, 0.6 m/s) now exhibiting more uniform grain structures and fewer unbonded regions. Porosity remains present in high-energy (100 W) samples, particularly at low scanning speeds (0.2 m/s, Figure 12g) due to gas entrapment. Some pores have coalesced or expanded (Figure 12g,h, 100 W, 0.2–0.4 m/s). Grain refinement and structural homogenization occur as heat treatment promotes grain growth, transforming columnar grains into a more equiaxed structure, reducing anisotropy. 100 W, 0.4–0.6 m/s samples (Figure 12h,i) exhibit the most refined microstructure, with reduced porosity and irregular grain growth. In summary, annealing significantly reduces lack of fusion defects, distributes porosity more uniformly in high-energy conditions (100 W, 0.2–0.4 m/s), and enhances isotropic expansion behavior, improving mechanical properties.

Based on the experimental conditions of this study and literature review, the phase transformation can likely be described below. In LPBF-fabricated Ti-6Al-4V, the as-built microstructure predominantly consists of a hierarchically organized prior-β grain structure filled with acicular martensitic α′ (hcp) laths. This non-equilibrium α′ phase leads to high strength but diminished ductility and contributes to atypical CTE behavior during initial heating. Upon annealing, decomposition of α′ into a more stable α + β lamellar microstructure begins near ~700 °C [37]. The nucleation of β phase typically occurs along α′ lath boundaries and at crystalline defects, while the morphology of lamellae evolves through diffusion-controlled coarsening [37]. Additionally, annealing promotes grain boundary migration, recrystallization, and potential globularization, phenomena that enhance microstructural isotropy and smooth out the CTE response [38]. These combined effects, phase redistribution, stress relief, and microstructural homogenization, explain the observed increase in CTE and its stabilization post-annealing.

CTE behavior of LPBF Ti-6Al-4V is also strongly influenced by microstructural defects, balling, lack-of-fusion, and porosity, observed in SEM (surface) and optical microscopy (cross-sections of as-fabricated and annealed samples). These defects drive differences between as-fabricated and annealed states and across laser powers (50, 75, 100 W) and scan speeds (0.2–0.6 m/s). Lack-of-fusion dominates under low energy: at 50 W (Figure 11a–c and Figure 12a–c) insufficient input causes weak interlayer bonding and voids, lowering CTE (Figure 9) and increasing scatter. At 75 W (Figure 11d–f and Figure 12d–f), lack-of-fusion is reduced and CTE becomes more stable. Annealing drives diffusion-based healing, strengthens interlayer bonds, and raises CTE, most notably in previously defect-prone conditions (50 W, 0.6 m/s). High-energy builds (100 W, 0.4 m/s; Figure 12h) already achieve effective fusion and thus show the most uniform CTE. Balling similarly degrades CTE stability under low-energy/high-speed settings (50 W, 0.2–0.6 m/s; Figure 10a–c), where melt-pool instability and surface-tension effects create irregular microstructures and density variations that cause CTE fluctuations. After annealing, microstructural refinement mitigates balling, yielding more uniform expansion. CTE stabilizes especially in samples that initially exhibited pronounced balling. Porosity follows the same trend, decreasing with higher energy and after annealing, supporting the observed increases in CTE and stability. Porosity strongly influences the CTE of LPBF Ti-6Al-4V, particularly under high energy input. In the as-built condition, keyhole-induced porosity is most pronounced in the 100 W, 0.2–0.4 m/s samples (Figure 10g,h, Figure 11g,h and Figure 12g,h), where excessive vaporization and keyhole instability promote pore formation. These pores reduce and destabilize CTE values, as entrapped gas pockets hinder uniform expansion. After annealing, some porosity remains, particularly in high-energy samples (Figure 11g,h and Figure 12g,h), but the pores become more evenly distributed, reducing their disruptive effect on expansion. CTE values improve after heat treatment, though they remain slightly lower than non-porous conditions due to the presence of residual voids limiting material expansion.

### 3.4. Comparison of CTEs of LPBF AlSi10Mg, 316L Stainless Steel, and Ti-6Al-4V Alloy

The CTE behavior of LPBF AlSi10Mg, 316L stainless steel, and Ti-6Al-4V alloys varies significantly due to differences in their material compositions, microstructures, and defect formations during processing. The CTE trends observed across these alloys are strongly influenced by laser power, scanning speed, and post-annealing treatment.

AlSi10Mg has the highest CTE due to its aluminum-based composition, but porosity and microstructural inhomogeneities impact stability, especially at low energy input. 316L stainless steel shows moderate and stable CTE, with anisotropic expansion in the as-fabricated state that improves with grain coarsening after annealing. Ti-6Al-4V has the lowest CTE, with annealing promoting equiaxed microstructure and better isotropic expansion. Increasing laser power enhances fusion and reduces porosity, leading to more stable CTE values. In AlSi10Mg, 50 W processing results in porosity and balling, while 100 W improves melt pool stability, yielding uniform expansion. 316L stainless steel is less affected by laser power changes due to its dense microstructure. Ti-6Al-4V shows the most significant response to power variation; 50 W leads to lack of fusion defects, while 100 W introduces keyhole-induced porosity, though with a lesser impact than in AlSi10Mg. Higher scanning speeds (0.6 m/s) generally lower CTE due to increased porosity and incomplete fusion. In AlSi10Mg, porosity-driven CTE reductions occur at high speeds, while 0.4 m/s balances melt efficiency and defect minimization. 316L stainless steel shows minor CTE variations with speed changes due to its dense structure. Ti-6Al-4V has unstable CTE at high speeds due to melt pool instability, but annealing helps homogenize the microstructure. Annealing improves CTE uniformity across all alloys by reducing defects and refining grain structures. In AlSi10Mg, annealing lowers CTE due to Si-rich precipitate formation and microstructural refinement. 316L stainless steel undergoes minimal change, though anisotropy decreases as columnar grains transform into an equiaxed structure. Ti-6Al-4V exhibited a slight increase in the CTE post-annealing owing to grain growth, which improved isotropic expansion.

The differences in CTE behavior between AlSi10Mg, 316L stainless steel, and Ti-6Al-4V alloys highlight the role of microstructural evolution in determining thermal expansion characteristics. AlSi10Mg, being an Al-based alloy, has inherently high thermal expansion but is significantly affected by processing defects like porosity and balling effects. 316L stainless steel exhibits a more stable expansion behavior, with minimal CTE variations between as-fabricated and annealed states. Ti-6Al-4V, on the other hand, demonstrates the lowest CTE due to its titanium-based composition and phase transformations during processing and annealing.

The results suggest that optimizing laser power and scanning speed is crucial in achieving desirable CTE properties for different applications. For AlSi10Mg, higher laser power (100 W) and moderate scanning speeds (0.4 m/s) yield the most consistent CTE behavior. For 316L stainless steel, CTE stability is less affected by processing parameters, making it a suitable material for applications requiring minimal thermal expansion fluctuations. For Ti-6Al-4V, optimizing heat treatment is particularly important, as annealing significantly enhances thermal expansion consistency by refining the grain structure and reducing anisotropic effects. The clear comparison between the CTEs behaviors of AlSi10Mg, 316L stainless steel, and Ti-6Al-4V alloy are shown in Table 3.

## 4. Conclusions

This study, for the first time, systematically investigated the CTE behavior of LPBF-fabricated AlSi10Mg, 316L stainless steel, and Ti-6Al-4V alloys under varying laser power, scanning speed, and annealing conditions. The results show that processing parameters and microstructural evolution strongly influence thermal expansion.

AlSi10Mg exhibited the highest CTE due to its aluminum-based composition, but was highly sensitive to porosity and balling defects at low energy inputs. 316L stainless steel maintained moderate and stable CTE values, with little change after annealing because of its inherently dense microstructure. Ti-6Al-4V showed the lowest CTE, with annealing slightly increasing its expansion through microstructural homogenization.

Optimal stability was generally achieved with 100 W laser power, 0.4 m/s scanning speed, and annealing, which together minimized defects and improved microstructural uniformity.

From a practical perspective: (i) AlSi10Mg at moderate power and scan speed provides stable CTE up to ~300 °C, making it suitable for electronic housings, optical mounts, and aerospace structures requiring dimensional precision. (ii) 316L stainless steel is reliable for cyclic thermal environments, though stress-relief annealing is recommended for applications such as marine or chemical components. (iii) Ti-6Al-4V is valuable for high-temperature aerospace and biomedical uses, but its non-linear CTE response near the α→β transition (~700 °C) must be considered in design.

These findings not only clarify the CTE behavior of three widely used LPBF alloys but also provide actionable guidance for parameter selection in applications demanding thermal reliability and dimensional stability.

## Figures and Tables

**Table 1:** Combinations of samples.

Variations in Samples	Laser Power (W)	Laser Scan Speed (m/s)	VED (J/mm^3^)
1	50	0.2	200
2	50	0.4	100
3	50	0.6	66.7
4	75	0.2	300
5	75	0.4	150
6	75	0.6	100
7	100	0.2	400
8	100	0.4	200
9	100	0.6	133.3

**Table 2:** Annealing heat treatment profiles for the alloys.

Alloy	Heating Rate (°C/min)	Temperature (°C)	Holding Time (min)	Cooling Strategy
AlSi10Mg	10	415	90	Cool within the furnace
SS 316L		700	90	
Ti-6Al-4V		785	90	

**Table 3:** Comparison of the Coefficient of Thermal Expansion (CTE) behavior of LPBF AlSi10Mg, 316L Stainless Steel, and Ti-6Al-4V alloys.

Aspect	AlSi10Mg	316L Stainless Steel	Ti-6Al-4V
Typical CTE Trend	Highest among the three alloys	Moderate and stable	Lowest among the three alloys
CTE Stability	Highly affected by porosity and balling	Least affected by defects due to dense structure	Fluctuates due to microstructural anisotropy
Effect of Annealing	Reduces CTE	Minimal change, improves isotropy	Increases CTE
Key Defects	Porosity, balling effect	Fine columnar grains, minor porosity	Lack of fusion, keyhole porosity
Best Processing Parameters	100 W, 0.4 m/s with annealing for uniform expansion	75 W, 0.4 m/s with annealing for homogeneity	100 W, 0.4 m/s with annealing for stability

## Data Availability

The original contributions presented in this study are included in the article. Further inquiries can be directed to the corresponding author.
